# VisProDom: an interactive Shiny/R application for displaying protein domains with transcriptional features

**DOI:** 10.1186/s12864-022-08713-3

**Published:** 2022-06-27

**Authors:** Hongwei Wang, Xiaotian Zhang, Shuangcheng Ding, Yujie Huang, Shengyong Wang, Huili Chen, Yuhang Chen, Yuting Li

**Affiliations:** 1 Hubei Key Laboratory of Waterlogging Disaster and Agricultural Use of Wetland/Engineering Research Center of Ecology and Agricultural Use of Wetland, Ministry of Education, Yangtze University, Jingzhou, 434000 China; 2 Hubei Collaborative Innovation Center for Grain Industry, Yangtze University, Jingzhou, 434000 China

**Keywords:** Protein domain, Transcript structure, R package, Shiny application, Visualization

## Abstract

**Background:**

Both the protein domains and transcript structures influence protein functional variation. The genomic location of both protein domains and transcript structural features can be described using the genomic coordinates of their encoded sequences. However, the coordinates of protein domains and transcriptional features often differ greatly, and it is difficult to view them in combination at the genome-wide level. In this paper, we describe the development of a new tool that allows users to visualize domains and transcript features together, using either built-in or uploaded genome datasets, and export publication-ready figures.

**Results:**

We developed a user-friendly, independent R package and Shiny web application named “VisProDom”. VisProDom consists of a genome-wide database containing entire annotated transcripts merged with annotated protein domains from the Pfam database. The built-in dataset includes 82 files, which merge genome general feature format (GFF) annotations with rpsblast tabular outputs from protein sequence searches in the Pfam database. Multiple genomes can be simultaneously screened for protein domains or transcript names. VisProDom includes step-by-step introductions and clickable elements for ease of use.

**Conclusion:**

VisProDom can display hundreds of transcripts alongside protein domains and export figures in a publication-ready format. This makes it a valuable tool for molecular evolution and comparative genomics.

**Supplementary Information:**

The online version contains supplementary material available at 10.1186/s12864-022-08713-3.

## Background

Genetic information can be characterized by both the protein domains and transcript structure. Protein domains are distinct functional and/or structural units that are usually responsible for a particular function or interaction that contributes to the overall role of the protein. Transcriptional features refer to features such as untranslated regions (UTRs), exons, coding sequences (CDSs), and introns. Genomic locations (i.e. coordinates) of protein domains are typically identified from protein sequence queries in the Pfam databases [1], the Simple Modular Architecture Research Tool (SMART) [2], and/or the Conserved Domain Database (CDD) [3]. It is important to understand the combination of transcript structure and protein domain at a genome-wide level, as it will help in identifying alternative splicing combinations and protein functional variations, and provide support for gene variation studies in important crop traits and major diseases. However, formulating a system for viewing transcript structures in combination with protein domains has been challenging. This is because transcripts are annotated at the genome level, whereas protein domains are annotated at the level of their corresponding transcript features. To overcome this, several tools have already been released [4–9]. An ideal tool for displaying protein domains should 1) include a function to create a database containing all annotated transcripts that are merged with protein domains; 2) allow users to run a genome-wide search of the database using the protein domain or transcript name; 3) build a large collection of files prior to merging transcriptional features with protein domains, and 4) allow users to build an independent database that can be used by the program. The tools currently available are insufficient, since none fulfill all four of these requirements. For example, DomainViz requires user to upload raw protein sequences for analysis; DOG functions in DOG2 aim to prepare publication-quality figures of protein domain structures without transcript features; Proter aims to visualize proteoforms and interactively integrate annotated and predicted sequence features together with experimental proteomic evidence; GSDS2.0, Mydomains, and transcript structure and domain display require users to input locations of features manually. We aimed to create a software package and web application that fulfills all four of the above requirements, thus creating an ideal tool for viewing transcript structures in combination with protein domains.

We designed VisProDom, a user-friendly, independent package for R, which is a widely used programming language for statistical computing and graphics. Additionally, we created a web application using Shiny (http://CRAN.R-project.org/package=shiny), an R (http://CRAN.R-project.org) package used to easily build interactive web applications.

## Implementation

Imported R packages used for the creation of VisProDom include ggplot2 (https://CRAN.R-project.org/package=ggplot2), Shiny (https://CRAN.R-project.org/package=shiny), rintrojs (https://CRAN.R-project.org/package=rintrojs), dplyr (https://CRAN.R-project.org/package=dplyr), data.table (https://CRAN.R-project.org/package=data.table), and plotly (https://cran.r-project.org/web/packages/plotly/index.html). The source code for VisProDom is freely available (https://github.com/whweve/VisProDom). We also deployed VisProDom online (https://whweve.shinyapps.io/VisProDom). Users can download and install VisProDom onto a local computer from GitHub by copying and pasting commands onto their R console. Owing to the characteristics of R and Shiny, VisProDom is a package that can be used in any operating system, requiring very limited programming skills.

## Data input

VisProDom contains 82 built-in genomes (Supplementary Table 1), annotated using the Pfam database. Users can also upload their own genome data. The details about how to access and upload datasets are described in the Workflow section. Users can upload genomes annotated using databases other than Pfam, and these can be compared to Pfam-annotated genomes. The R function VisProDOM::CreDat is used to merge genome files with protein annotation files. Two input files are necessary for the R function VisProDOM::CreDat; the general feature format (GFF) file and the protein annotation file. Protein annotation files can be obtained by a query of the transcript protein sequence against a domain database such as Pfam, SMART, or CDD, using the rpsblast tool [10]. With the help of VisProDOM::CreDat, users can expand the scope of genome and protein annotation in VisProDom by incorporating data from other databases. With this tool, users can select the required database to enhance. Users may simultaneously view multiple genomes annotated using a variety of protein domain databases.

## Workflow

To use the VisProDom R package, R (Comprehensive R Archive Network) and RStudio (an integrated development environment for R) have to be downloaded. Currently, the source code of VisProDom is hosted on GitHub (https://github.com/whweve/VisProDom). The R package, “devtools”, is required to download and install VisProDom from GitHub. VisProDom requires other R packages. When launching RStudio, the required packages and VisProDom can be installed and loaded using the following commands in the R environment:

-  > install.packages(c(“devtools”, “ggplot2”, “reshape2”, “dplyr”, “shiny”, “fresh”, “rintrojs”, “data.table”, “markdown”)).
-  > devtools:install_github(“whweve/VisProDom”).
-  > library(VisProDom).
- >  runVPDapp()

To use VisProDom without installing it into the local computer environment, the Shiny-based web application can be used (https://whweve.shinyapps.io/VisProDom/). The web application can be accessed from most internet browsers, including Firefox (https://www.mozilla.org), Microsoft Edge (https://www.microsoft.com/en-us/edge), and Chrome (https://chrome.en.softonic.com).

**Fig. 1:**
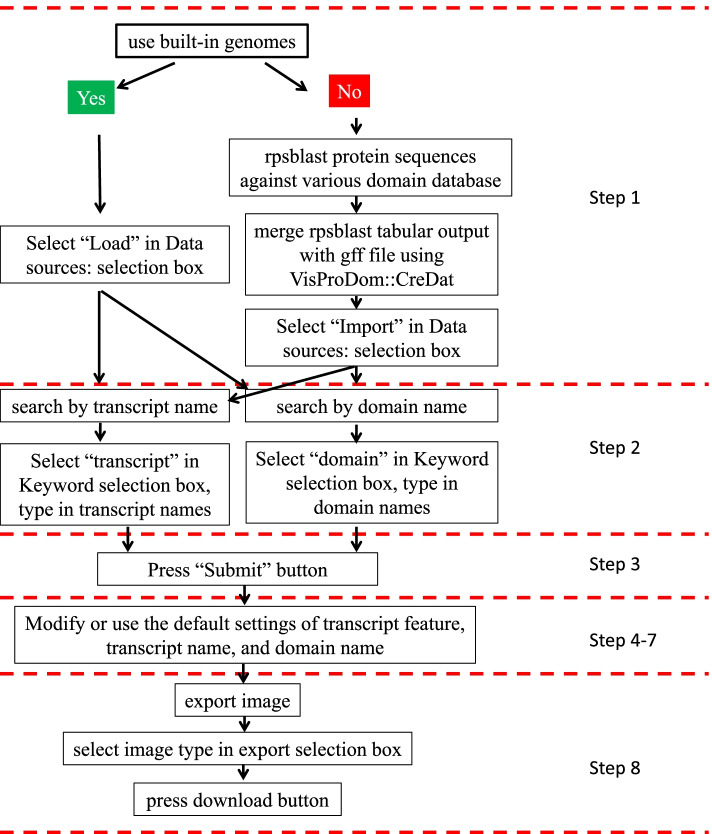
The workflow for VisProDom, a Shiny/R application for displaying protein domains and transcript structure of built-in or user-uploaded genomes

Both versions of VisProDom include a workflow of eight steps (Fig. 1).

In step one, the user selects one or more built-in or self-uploaded genomes. To use built-in genomes, the user selects the “Load” option in the “Database” field and then the desired genome from the “Genome” field. To upload new genome data, the user selects the “Import” option in the “Database” field. The user will be prompted to tell VisProDom how many genomes will be imported via the “inputted gff number” field. When the user clicks on “Browse,” a dialogue box will appear, allowing the user to select and upload each relevant file.

In step two, the user queries their selected genomes for transcript features or domains. Domain names can be queried using one of three qualifying expressions: 1) one or more entered domains regardless of the background domain, 2) all entered domains regardless of the background domain, or 3) all entered domains with no background domain. When complete, the user clicks on the “Update VisProDom” button.

In steps three to seven, users can manually adjust visual elements of their generated figure, such as the color and size of text and transcript features. However, this is not necessary since these features are auto-generated to be in proportion to the number of transcripts visualized.

Finally, in step eight, users can adjust the file format, size, and resolution as required, and export their generated figures. VisProDom provides step-by-step instructions and clickable elements created using the R package “rintrojs”, making it easy to use.

## Results and discussion

VisProDom is an easy-to-use stand-alone R package and Shiny-based web application that displays domains and transcripts using built-in genomes. The VisProDom R package can be downloaded and installed from GitHub (https://github.com/whweve/VisProDom), following the instructions on the web page. The Shiny web application can be accessed online (https://whweve.shinyapps.io/VisProDom) with no installation needed.

**Fig. 2:**
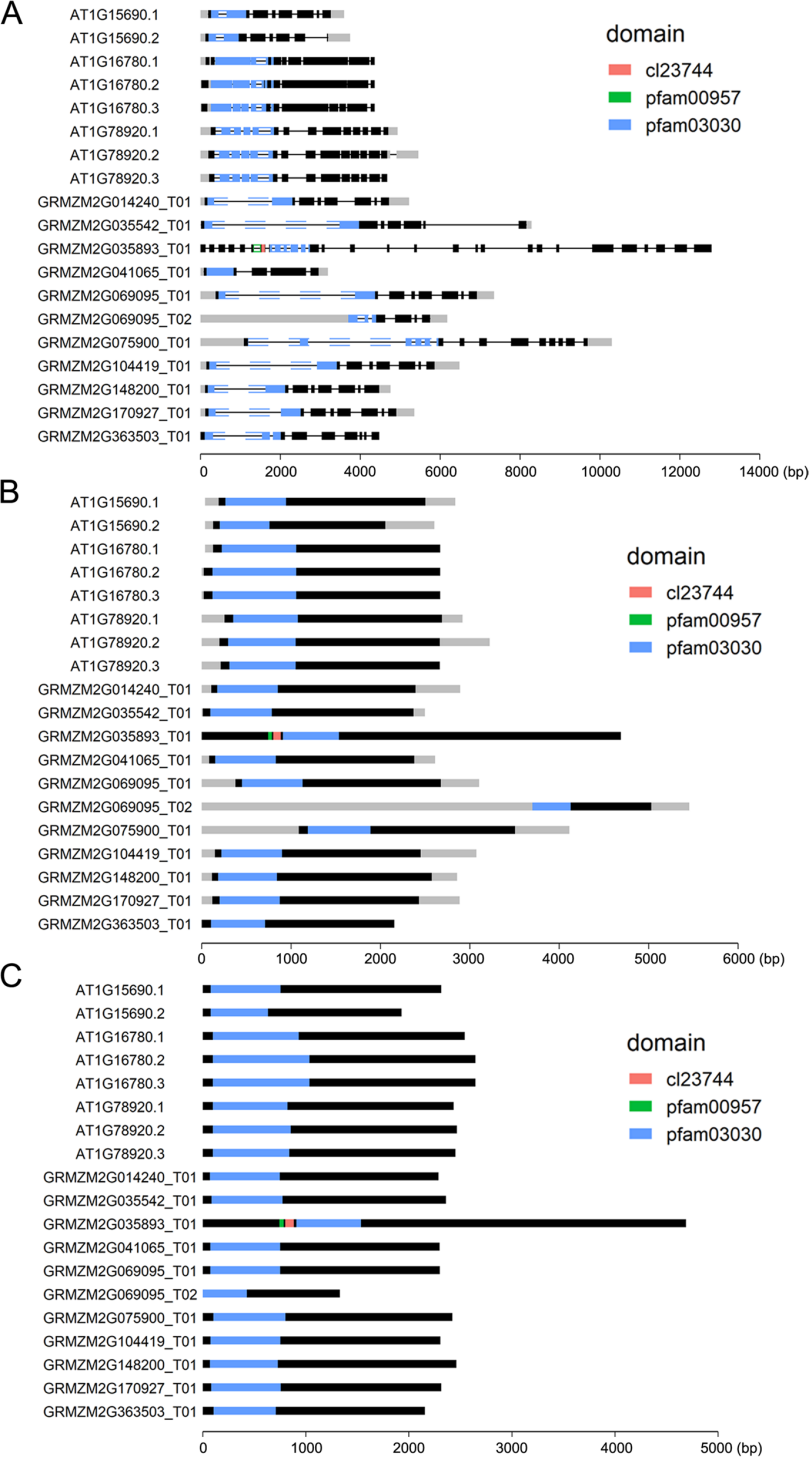
The output of protein domain Pfam08774 search against maize B73 (genome version: AGP_V3) and Arabidopsis thalilian (genome version: araport11.447) genomes using the VisProDom R package, showing **a**) protein domain with transcript feature; **b**) protein domain with cDNA feature, and **c**) protein domain with CDS feature. cDNA, complementary DNA; CDS, coding sequence

VisProDom can visualize hundreds of transcripts in a publication-ready format. Furthermore, it will generate figures with the font size of transcript names automatically adjusted according to the number of transcripts. To show an example output, we searched the protein domain Pfam08774 against the maize B73 genome (Genome version: AGP_V3) and *Arabidopsis thalilian* genome (Genome version: araport11.447), irrespective of the background domain (Fig. 2).

In comparison to existing tools [4–6], VisProDom is more suitable for performing multiple domain searches with regular expression patterns across multiple genomes at the genome-wide level. The selection of multiple genomes enables the comparison of transcript structure and domain distribution between species. This makes it a convenient method for identifying evolutionary relationships.

Currently, VisProDom does not include phylogenetic tree data, and transcript sequences are not ordered according to phylogenetic relationship. However, in the future we aim to expand the capabilities of VisProDom to include phylogenetic data, and extend its applicability in the field of evolutionary biology.

## Conclusions

VisProDom is capable of visualizing hundreds of transcripts and domains across multiple selected species in a publication-ready format, making it a valuable tool for molecular and evolutionary biology. The built-in genome data improves usability; since many entire genomes are readily available, users are not required to enter protein sequences, and the running speed is high. Its ease of use is further improved by the provision of detailed instructions within the application. While VisProDom is available as an R package, its availability as a Shiny-based web application ensures that it is highly accessible as a platform-free tool.

## Supplementary Information

**Additional file 1:** **Supplementary Table 1.** The 82 genomes built in to VisProDom. 

## Data Availability

VisProDom source code and tutorial are freely available at https://github.com/whweve/VisProDom. The online VisProDom Shiny application is freely available at https://whweve.shinyapps.io/VisProDom/.

## Abbreviations

CDD: Conserved Domain Database
CDS: Coding sequence
GFF: General feature format
SMART: Simple Modular Architecture Research Tool
UTR: Untranslated region
